# Small RNAs as systemic signals in plant defense: mechanisms, challenges, and future directions

**DOI:** 10.1007/s11033-026-11817-8

**Published:** 2026-04-16

**Authors:** Clabe Wekesa, Kelvin Kiprotich, John Muoma, Axel Mithöfer

**Affiliations:** 1 Research Group Plant Defense Physiology, Max Planck Institute for Chemical Ecology, Jena, Germany; 2 Department of Soil Science, Stellenbosch University, Stellenbosch, South Africa; 3 Department of Biological Sciences, Masinde Muliro University of Science and Technology, Kakamega, Kenya

**Keywords:** Systemic signaling, sRNAs, RNA mobility, Cross-kingdom RNAi, Host-induced gene silencing

## Abstract

Small RNAs (sRNAs) have emerged as central regulators of gene expression, coordinating development, stress responses, and immunity across plant tissues. Far from acting solely within individual cells, sRNAs move through plasmodesmata and the phloem to mediate systemic silencing, forming a long-distance communication network that parallels classical hormonal signaling. This review synthesizes current evidence for sRNA mobility in plants and its extension across species boundaries during host-pathogen interactions. We describe how sRNAs are stabilized by RNA-binding proteins, Argonaute complexes, and extracellular vesicles (EVs), and how these carriers enable selective trafficking within the plant and into fungal or oomycete pathogens. Cross-kingdom RNA interference (ckRNAi) forms the mechanistic basis of Host-Induced Gene Silencing (HIGS) and Spray-Induced Gene Silencing (SIGS), two emerging RNA-based strategies for crop protection. We also addressed the variability of RNA uptake among pathogens, environmental instability of naked RNAs, and the promise of nanocarriers, synthetic biology, and machine-learning design tools to overcome these barriers. Bioinformatic and regulatory challenges, ranging from the identification of functional mobile RNAs to risk assessment and field validation remain key frontiers. Collectively, these advances position mobile RNAs as both mechanistic signals and deployable tools, redefining plant-microbe communication and opening new paths toward predictive, sustainable RNA-driven agriculture.

## Introduction: systemic signaling in plant defense

 Plants are continuously exposed to a wide range of biotic stresses, including viruses, bacteria, fungi, oomycetes, nematodes, and insect herbivores. Because of their sessile lifestyle, they cannot escape these threats but instead rely on a finely tuned immune system that integrates local perception of attack with systemic communication to mount a whole-plant defense response [1, 2]. Systemic signaling is essential to activate resistance in tissues that have not yet been colonized, thereby restricting pathogen spread and reducing overall damage [3]. Classical systemic signals include phytohormones such as salicylic acid (SA), jasmonic acid (JA), and abscisic acid (ABA). These are central to both systemic acquired resistance (SAR) and induced systemic resistance (ISR) [4]. Peptide signals such as systemin and Pep peptides, reactive oxygen species, electrical signals, and hydraulic waves also contribute to rapid long-distance communication [5–9]. While these chemical and electrical signals have been studied for decades, an additional epigenetic layer has recently gained attention, sRNAs.

sRNAs are short non-coding RNAs, typically 18–30 nucleotides in length, that regulate gene expression post-transcriptionally through mRNA cleavage or translational repression or transcriptionally through chromatin modification and DNA methylation [10, 11]. They are broadly classified into microRNAs (miRNAs), which are processed from hairpin precursors, and small interfering RNAs (siRNAs), which often arise from double-stranded RNA [12]. In plant immunity, sRNAs regulate numerous defense-related genes, including those controlling receptor abundances, hormone signaling, and secondary metabolite pathways [13]. A landmark in the recognition of sRNAs as mobile signals was the discovery that miR399, which regulates phosphate homeostasis, travels through the phloem from shoots to roots to repress *PHO2* expression [14]. This finding established the paradigm that sRNAs can act as long-distance signals beyond their site of origin. Subsequent evidence has shown that stress-responsive sRNAs accumulate in vascular exudates and can move across tissues [15, 16]. In defense, miR393 restricted auxin signaling during *Arabidopsis*-*Pseudomonas syringae* interactions [17]. Moreover, systemic silencing triggered by siRNAs derived from viruses or transgenes demonstrated that sRNA-based information can spread through plants to establish broad-spectrum resistance [18]. Perhaps most striking is the phenomenon of RNAi, whereby sRNAs move between organisms during plant-pathogen or plant-pest interactions. Plants have been shown to export sRNAs into fungal pathogens [19] to suppress pathogenicity genes. Conversely, pathogens can deliver their own sRNAs into host plants to suppress host defense genes, effectively hijacking the host RNAi machinery [20, 21].

Despite these advances, several challenges remain. Unlike classical hormones, the mechanisms underlying sRNA mobility are only partially understood. Key mechanistic gaps concern how these molecules are selectively packaged into carriers such as extracellular vesicles, how they maintain stability during long-distance transport through the vasculature, and how specificity in target recognition is achieved in recipient tissues. Moreover, although numerous sRNAs have been catalogued in vascular exudates, only a few have been functionally linked to systemic defense outcomes. Most evidence still comes from *Arabidopsis* and model crops under controlled laboratory conditions, leaving open the question of whether sRNA-mediated signaling plays a comparable role in natural environments where multiple stresses interact.

At the translational level, the field faces equally pressing challenges. Synthetic sRNA sprays and host-induced gene silencing Host-Induced Gene Silencing (HIGS) approaches show considerable promise for crop protection, yet their field performance, delivery efficiency, and environmental stability remain limited [22]. In parallel, current sequencing and bioinformatic workflows introduce biases that hinder the detection and accurate quantification of rare but functionally important mobile sRNAs [23]. Addressing these mechanistic and translational constraints will be essential to move sRNA research from a largely descriptive framework toward predictive and application-oriented biology. The conceptual understanding of small RNAs in plant systems has also evolved substantially over time. Early studies established RNA silencing primarily as a local gene regulatory and antiviral mechanism. This view expanded with the discovery that specific small RNAs, such as miR399, function as long-distance signals coordinating nutrient homeostasis via vascular transport. Subsequent work demonstrated that mobile small RNAs can act as positional cues during developmental patterning, moving between tissues to establish spatial gene expression domains. More recently, these concepts have been extended to plant immunity, where small RNAs are now recognized as systemic signals and mediators of cross-kingdom communication with pathogens. These advances reflect a shift from viewing small RNAs as intracellular regulators to recognizing them as integral components of intercellular and long-distance signaling networks in plants. Several reviews have addressed the general roles of sRNAs in plant immunity [24, 25]. However, a focused synthesis that specifically examines small RNAs as systemic defense signals, integrating their mobility, regulatory mechanisms, and translational potential remains lacking. This review therefore aims to (1) summarize current evidence for sRNA mobility in plant defense, including both intra-plant and cross-kingdom contexts, (2) highlight key mechanistic and translational challenges that limit our understanding of sRNA-mediated signaling, and (3) outline future directions for harnessing sRNAs in crop improvement and sustainable plant protection.

## Functional overview of SRNAs in plant defense

The sRNAs constitute a diverse class of 18–30 nucleotide non-coding RNAs that regulate gene expression through sequence-specific silencing mechanisms [1, 12]. In plants, they act at both post-transcriptional and transcriptional levels, providing a powerful means of controlling development, metabolism, and defense [26]. The two main classes, miRNAs and siRNAs, share mechanistic similarities but differ in their origins, processing, and regulatory scopes. miRNAs are transcribed by RNA polymerase II as single-stranded pri-miRNA precursors that fold into stem-loop structures. In the nucleus, these are processed by DICER-LIKE 1 (DCL1) in association with cofactors HYPONASTIC LEAVES 1 (HYL1) and SERRATE (SE) into ~ 21–22 nucleotide miRNA/miRNA* duplexes, which are subsequently 2′-O-methylated by HUA ENHANCER 1 (HEN1) to prevent degradation [27]. The methylated duplex is exported to the cytoplasm by the Exportin 5 homolog, HASTY (HST), where the mature miRNA strand is selectively loaded into ARGONAUTE 1 (AGO1) to form the RNA-induced silencing complex (RISC). This complex mediates post-transcriptional gene silencing (PTGS) through target mRNA cleavage or translational repression.

**Fig. 1:**
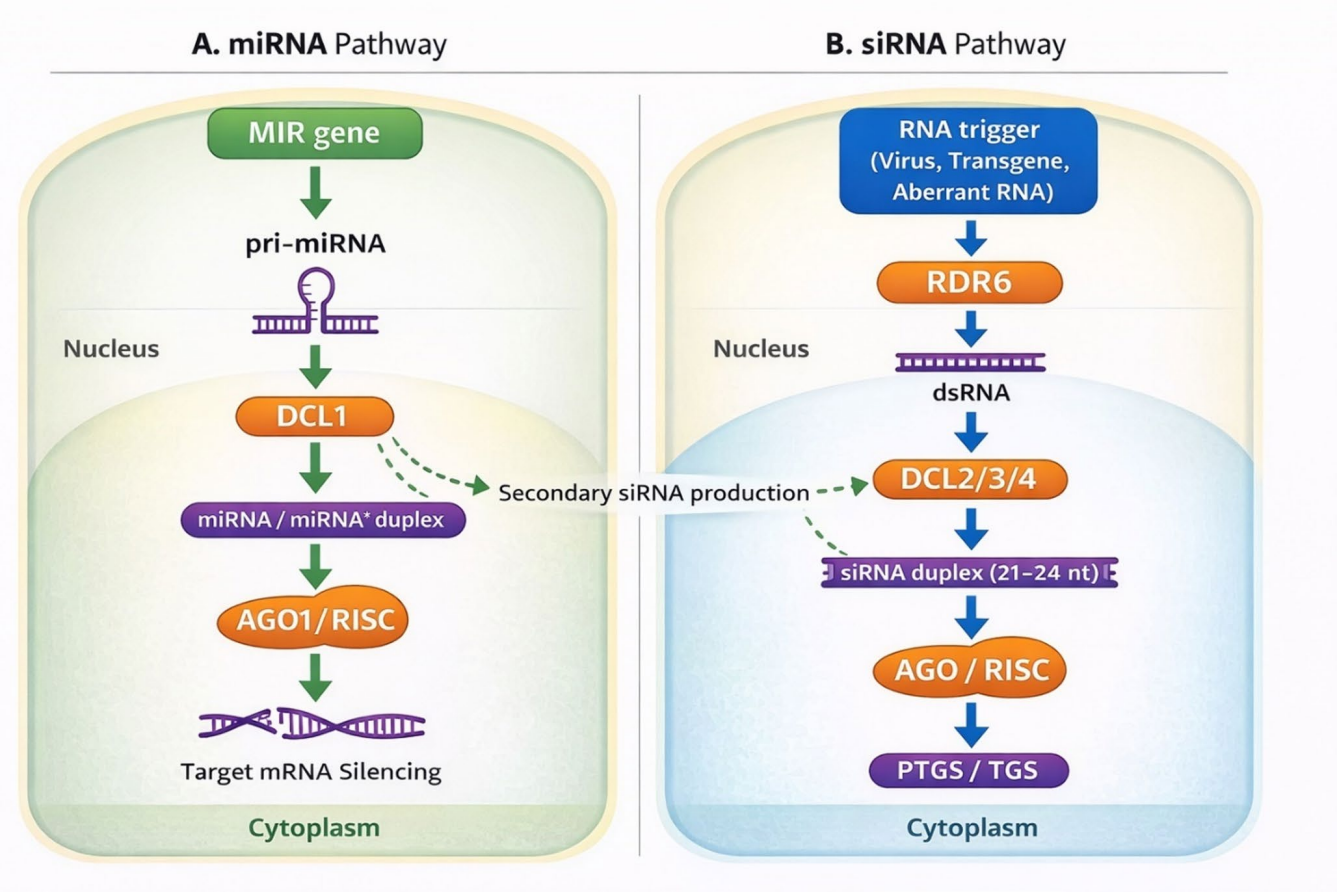
Biogenesis and mechanisms of miRNAs and siRNAs in plants. (**A**) In the miRNA pathway, MIR genes are transcribed by RNA polymerase II to produce pri-miRNA transcripts, which are processed by DCL1 in the nucleus and exported to the cytoplasm. The mature miRNA/miRNA duplex is loaded into AGO1-containing RNA-induced silencing complexes (RISC) to mediate post-transcriptional gene silencing (PTGS) through mRNA cleavage or translational repression. (**B**) In the siRNA pathway, RNA-dependent RNA polymerases (RDRs) generate double-stranded RNA (dsRNA), which is processed by DCL2/3/4 into siRNA duplexes. These siRNAs associate with AGO proteins to direct PTGS in the cytoplasm or RNA-directed DNA methylation (RdDM) and transcriptional gene silencing (TGS) in the nucleus. miRNA-mediated cleavage can also trigger secondary siRNA production

In contrast, siRNAs arise from double-stranded RNA (dsRNA) precursors generated by RNA-dependent RNA polymerases (RDRs) or other dsRNA-producing processes and processed by DCL2, DCL3, or DCL4 into 21-, 22-, or 24-nucleotide species [25, 28]. These siRNA duplexes are methylated by HEN1 and partition into two functional branches: 21–22 nt siRNAs associate with AGO proteins such as AGO1 and AGO2 in the cytoplasm to direct PTGS, whereas 24 nt siRNAs bind AGO4, AGO6, or AGO9 in the nucleus and recruit DOMAINS REARRANGED METHYLTRANSFERASE2 (DRM2) to promote RNA-directed DNA methylation (RdDM) and histone modification (Fig. 1). This process establishes transcriptional gene silencing (TGS) and long-term epigenetic regulation, ensuring stable repression of transposons and maintenance of genome integrity [29].

In the context of plant immunity, these small RNA pathways form an essential regulatory layer that fine-tunes receptor abundance, hormone signaling, and pathogen defense gene expression. Pathogen perception rapidly reprograms miRNA expression to optimize the balance between growth and immunity. A well-characterized example is miR393, which suppresses *TIR1/AFB* auxin receptors upon flagellin perception, dampening auxin signaling to enhance resistance against *Pseudomonas. Syringae*, bacterial speck pathogen [17]. Other miRNAs, including miR160 and miR167, similarly modulate auxin response factors to maintain homeostasis during infection [30]. miR398 integrates oxidative stress responses into immunity by targeting Cu/Zn superoxide dismutases, allowing the plant to redirect copper from metabolic enzymes toward defense-related cuproproteins under stress [31]. In addition to miRNAs, siRNAs play critical roles in antiviral defense and transposon silencing. Viral double-stranded RNAs are processed by Dicer-like 4 (DCL4) and DCL2 into 21- and 22-nt siRNAs, respectively. These siRNAs are subsequently loaded into ARGONAUTE 1 (AGO1) and related AGO proteins, which guide the sequence-specific cleavage of viral genomes and transcripts, forming the molecular basis of RNA interference (RNAi)-mediated antiviral immunity [32, 33]. The 24-nt heterochromatic siRNAs preserve genome stability by directing DNA methylation at transposon loci. This methylation silences transposable and other repetitive elements, preventing their activation during stress or viral infection [34].

A hallmark of the plant RNA silencing system is its self-amplifying nature. Primary siRNAs generated from initial target recognition can trigger RNA-DEPENDENT RNA POLYMERASE 6 (RDR6)-mediated synthesis of secondary siRNAs, extending silencing beyond the original locus and reinforcing systemic protection [35, 36]. This amplification property enables silencing signals to spread from locally infected tissues to distant, uninfected organs, a phenomenon termed systemic acquired silencing [18, 37]. Through this mechanism, sRNAs not only suppress pathogens at the site of attack but also prime distal tissues for subsequent defense, providing an RNA-based analogue of systemic acquired resistance. These silencing pathways are linked with hormonal and epigenetic networks. sRNA-mediated regulation intersects with SA, JA, and ethylene signaling, thereby determining the outcome of immune responses against biotrophic versus necrotrophic pathogens [24, 38]. In parallel, 24-nt siRNAs involved in RdDM can imprint stable epigenetic modifications on defense-related loci, reinforcing immune responses across developmental stages and, in some cases, across generations [39]. Such integration points to the versatility of sRNAs as both rapid-response mediators and long-term stabilizers of plant immune competence.

Beyond defense, mobile sRNAs also play pivotal roles in developmental patterning, acting as positional cues that coordinate gene expression across tissues. In *Arabidopsis* leaves, tasiR-ARFs, a class of trans-acting siRNAs derived from *TAS3* transcripts, move intercellularly from their biogenesis site on the adaxial (upper) side toward the abaxial (lower) side of the lamina. This directional movement establishes a gradient that spatially restricts the expression of the abaxial determinant *AUXIN RESPONSE FACTOR3* (*ARF3*), thereby defining leaf polarity [40]. Similarly, in roots, miR165/166 are synthesized in the endodermis but migrate inward into the stele, where they degrade *HD-ZIP III* transcripts in a dosage-dependent manner. The resulting gradient of target mRNA specifies protoxylem and metaxylem cell fates, demonstrating that miRNA mobility is essential for radial tissue organization [41].

## Current evidence of small rna mobility in defense

The following studies provide experimental evidence supporting the role of small RNAs as systemic signals in plant defense.

### sRNAs in vascular transport

The first indications that sRNAs function as systemic messengers came from their detection in vascular tissues, particularly phloem sap. Early analyses of phloem exudates from *Cucurbita maxima*,* Brassica napus*, and *Arabidopsis thaliana* revealed that phloem is enriched in a defined set of sRNAs, including mature miRNAs and siRNAs, rather than random degradation products [16, 42, 43]. Many of these phloem-resident sRNAs were stress-responsive, such as miR395, miR398, and miR399, which accumulate under sulfur, copper, and phosphate limitation, respectively, indicating that they act as long-distance informational signals linking local nutrient status to systemic responses. Importantly, only fully processed, single-stranded mature miRNAs were detected in the phloem, suggesting that systemic mobility is not due to leakage of precursor molecules but rather to selective stabilization and regulated export from source tissues [42]. This was a critical observation because it implied that plants invest in mechanisms to sort, package, and protect sRNAs for long-distance transport, rather than simply releasing cellular debris into the vascular stream.

Several sRNAs have now been experimentally confirmed to move between tissues or even across species boundaries, where they regulate nutrient signaling and defense **(**Table 1**).** These include miR399 and miR2111, which control phosphate uptake and nodule formation, respectively, and cross-kingdom mobile RNAs such as miR166 and miR159 from cotton that target fungal virulence genes in *Verticillium dahliae*,* the soil-borne fungus that causes Verticillium wilt*.

**Table 1:** Summary of experimentally validated mobile sRNAs and their targets in systemic signaling and defense

Mobile sRNA	Plant species / system	Source tissue → Destination	Confirmed target gene(s)	Biological function / pathway	Experimental evidence	References
miR399	*Arabidopsis thaliana*,* Brassica napus*	Shoot → Root (via phloem)	PHO2 (E2 ubiquitin-conjugating enzyme)	Phosphate homeostasis; systemic nutrient signaling	Grafting, qPCR, and reporter assays showing PHO2 repression in rootstocks	[15, 44]
miR2111	*Lotus japonicus*,* Medicago truncatula*	Shoot → Root	TML (Too Much Love, nodulation repressor)	Long-distance regulation of nodule formation	Phloem sap sequencing and grafting analysis	[45]
miR395	*B. napus*,* A. thaliana*	Shoot ↔ Root	APS, SULTR2;1	Sulfate assimilation and remobilization under sulfur limitation	Phloem exudate profiling; nutrient starvation assays	[46]
miR398	*A. thaliana*	Shoot ↔ Root, stress-responsive	CSD1, CSD2 (Cu/Zn-superoxide dismutases)	Redox homeostasis and oxidative stress response	Mutant and overexpression analyses under copper limitation	[24, 31]
miR166 / miR159	*Gossypium hirsutum → Verticillium dahliae*	Host → Pathogen (cross-kingdom)	Clp-1, HiC-15	Silencing of fungal virulence genes; host-induced defense	Detection of plant miRNAs in fungal hyphae; functional complementation assays	[19]
siRNAs (vsiRNAs)	*Nicotiana benthamiana*	Infected leaf → Upper leaves	Viral genomic RNAs	Systemic antiviral silencing	Grafting and deep sequencing of mobile siRNAs	[18, 47]
sRNAs in extracellular vesicles EVs	*A. thaliana → Botrytis cinerea*	Host → Pathogen	Bc-VPS51, Bc-DCL1, Bc-DCL2	RNA-mediated suppression of fungal pathogenicity	Isolation of EVs enriched in sRNAs; target cleavage validation	[21, 48]
22-nt miRNAs	*Cuscuta campestris → A. thaliana*	Parasite → Host	Multiple defense-related genes	Parasitic manipulation of host transcriptome	AGO1 immunoprecipitation; degradome mapping	[49]

In *Arabidopsis*, phosphate starvation induces the accumulation of miR399 in shoots. The mature miRNA is then transported through the phloem to the roots, where it represses the ubiquitin-conjugating enzyme PHO2, a negative regulator of phosphate uptake. Grafting experiments provided direct evidence for its mobility: mature miR399 accumulated to high levels in wild-type rootstocks grafted to miR399-overexpressing scions, while the primary transcripts remained confined to the shoots [44]. A similar long-distance signaling role has been described for miR2111 in legumes. In *Lotus japonicus*, shoot-derived miR2111 moves via the phloem to the roots, where it suppresses the nodulation repressor TOO MUCH LOVE (*TML*), maintaining root susceptibility to symbiotic infection [45].

Consistent with these examples, analyses of *B. napus* vascular exudates revealed that RNA is absent from the xylem but abundant in the phloem, which contains numerous sRNAs including mature miRNAs [42]. Among these, miR395, miR398, and miR827 are selectively enriched under sulfate, copper, and nitrogen limitation, respectively, highlighting the phloem as a central route for nutrient-responsive sRNA transport. During sulfate starvation, phloem-mobile miR395 represses ATP sulfurylase *(APS)* and the low-affinity sulfate transporter *SULTR2;1* in shoots, thereby restricting local assimilation and promoting sulfate remobilization to roots and developing tissues [46]. Under copper limitation, miR398 targets Cu/Zn-superoxide dismutases (*CSD1* and *CSD2*), conserving copper for essential photosynthetic cuproproteins and maintaining redox balance [24]. Likewise, miR827, detected in phloem sap, targets *NLA* (Nitrogen Limitation Adaptation) to regulate phosphate transporter turnover, thereby integrating nitrogen and phosphate homeostasis.

Although the phloem is a highly oxidative and RNase-rich environment, sRNAs maintain remarkable stability during long-distance transport. This protection is achieved through their association with RNA-binding proteins (RBPs), Argonaute (AGO) complexes, and possibly extracellular vesicles (EVs). In *Arabidopsis*, AGO1 and AGO2 have been detected in phloem exudates, where they likely shield bound miRNAs from degradation and guide them to recipient cells [42, 50]. Other abundant phloem RBPs, such as PSRP1, CmPP16, and phloem small RNA-binding protein 1 (PSRP1), form ribonucleoprotein (RNP) complexes that facilitate sRNA stability and mobility [51, 52]. More recently, the identification of vesicle-like nanoparticles and EV markers (TET8, ANN1/2, HSP70) in phloem sap suggests that a subset of sRNAs may be packaged into vesicles for selective, protected transport [53]. In addition to local RNA silencing, several studies have demonstrated that small RNAs can move systemically within plants to coordinate defense responses.

### Systemic silencing and within-plant defense cases

Beyond nutrient signaling, sRNAs are central to systemic defense communication in plants, forming part of a molecular network that coordinates immune responses between local and distal tissues. Upon pathogen attack, plants initiate a rapid and localized burst of sRNA production, followed by long-distance movement of these molecules to prime uninfected regions, a process often referred to as systemic acquired silencing (SAS) [54]. Unlike SAR, which is primarily mediated by phytohormones such as salicylic acid, SAS is driven by mobile small RNAs that induce sequence-specific gene silencing. This layer of defense complements systemic acquired resistance (SAR) mediated by salicylic acid, highlighting the deep integration between hormonal and RNA-based signaling pathways.

A prominent example is miR393, one of the earliest miRNAs shown to respond to pathogen perception. Following recognition of the bacterial flagellin peptide flg22 by the FLS2 receptor, *Arabidopsis* induces miR393, which targets transcripts encoding the auxin receptors *TIR1* and *AFB1/2/3* [17]. By repressing auxin signaling, miR393 shifts cellular priorities from growth to defense, limiting pathogen entry and multiplication. This exemplifies how miRNAs rapidly reprogram hormonal networks to reinforce immunity. Other auxin-related miRNAs, such as miR160 and miR167, target *ARF* transcription factors and further modulate auxin responses during stress [55]. Likewise, miR398 plays a crucial redox-protective role by modulating the balance between oxidative stress responses and growth. Under normal conditions, miR398 maintains low basal levels of its targets, the Cu/Zn-superoxide dismutases *CSD1* (cytosolic) and *CSD2* (chloroplastic), thereby fine-tuning their spatial and temporal expression. However, during oxidative or high-light stress, miR398 transcription is repressed, leading to the accumulation of *CSD1* and *CSD2* transcripts, which enhance the detoxification of superoxide radicals [31].

Systemic spread of silencing signals was first demonstrated through elegant transgenic and grafting experiments. Early studies using fluorescent reporter plants showed that silencing of a transgene in one leaf could trigger sequence-specific repression in distant leaves, implying the movement of a mobile silencing factor [56, 57]. The molecular nature of this factor was clarified by [18], who proved that siRNAs themselves, not proteins constitute the transmissible silencing signal. In their study, 21-nt siRNAs generated locally spread through the vasculature to silence homologous GFP transgenes in remote tissues, while 24-nt siRNAs promoted transcriptional silencing through RNA-directed DNA methylation (RdDM). These findings established that the size and biochemical identity of siRNAs dictate the layer of gene control: 21–22 nt species mediate post-transcriptional gene silencing (PTGS), whereas 24 nt siRNAs induce transcriptional gene silencing (TGS) via epigenetic modification. Further studies revealed that systemic silencing involves both cell-to-cell spread through plasmodesmata and long-distance transport through the phloem stream [50]. RNA-binding proteins such as SDE5, AGO1, and RDR6 participate in the amplification and stabilization of these mobile silencing signals. Once delivered to distal tissues, mobile siRNAs guide AGO complexes to homologous transcripts or chromatin regions, ensuring that silencing patterns are reproduced far from the original infection site. This mobility enhances the plant’s capacity for rapid systemic defense responses to subsequent pathogen challenges.

The biological relevance of systemic RNA movement extends beyond artificial transgenes. In natural infections, virus-derived siRNAs (vsiRNAs) generated in infected leaves can travel through the phloem to non-infected tissues, where they direct cleavage of viral genomes or transcripts and prevent systemic viral spread [58]. For example, in *N. benthamiana*, vsiRNAs corresponding to *Tobacco mosaic virus* sequences were detected in upper, uninfected leaves, correlating with strong antiviral immunity [47]. Similarly, *Cucumber mosaic virus* and *Potato virus X* infections induce siRNA accumulation in distant organs, providing compelling evidence that systemic silencing constitutes a natural antiviral surveillance system [59]. This RNA-mediated systemic defense signaling operates analogously to adaptive responses, but through sequence homology rather than somatic recombination.

In addition to viruses, mobile siRNAs also participate in defense against endogenous genomic threats. The 24-nt heterochromatic siRNAs produced by DCL3 and loaded into AGO4 or AGO6 travel locally and, in some cases, systemically to reinforce transcriptional silencing of transposons and repetitive DNA elements safeguarding genome integrity under stress [34]. These siRNAs guide DOMAINS REARRANGED METHYLTRANSFERASE 2 (DRM2) to direct cytosine methylation and deposition of repressive histone marks such as H3K9me2 at target loci, establishing heterochromatin and coupling RNA interference to stable epigenetic inheritance [29]. Similar mobile 24-nt siRNAs have been observed during paramutation in maize, where siRNA-mediated trans-allelic communication maintains heritable gene silencing between homologous loci [60]. In *Arabidopsis*, stress conditions such as heat or salinity can trigger redistribution of heterochromatic siRNAs between somatic tissues and reproductive cells, ensuring that transposon silencing and genome stability are preserved in the germline [61, 62]. Moreover, siRNAs generated in companion or nurse cells can move into gametes to reactivate RdDM in zygotic tissues, exemplifying intercellular reinforcement of epigenetic control [63]. Systemic silencing establishes an integrated network of genome defense which extends beyond immediate pathogen responses to long-term maintenance of genomic integrity.

The systemic spread of RNA silencing relies on two key processes: signal amplification and intercellular trafficking. Local silencing is reinforced by RNA-Dependent RNA Polymerase 6 (RDR6) and its cofactor SUPPRESSOR OF GENE SILENCING 3 (SGS3), which convert target mRNAs into double-stranded RNA templates that are subsequently diced into secondary siRNAs [64–66]. This amplification enables the generation of abundant 21- and 22-nt siRNAs that sustain silencing even as primary triggers decay. These secondary siRNAs can move from cell to cell through plasmodesmata, the cytoplasmic channels that interconnect plant cells. The extent of movement is modulated by plasmodesmata-gating proteins, including callose synthases and β-1,3-glucanases, which dynamically adjust channel permeability during stress or infection [67–69]. Such regulated intercellular trafficking allows silencing signals to spread from initially infected or transformed cells to neighboring tissues and ultimately into the vascular system, where mobile siRNAs are loaded for long-distance transport. Beyond intra-plant signaling, small RNAs can also move across species boundaries, mediating ckRNAi during plant-pathogen interactions.

### Cross-kingdom RNA interference

**Fig. 2:**
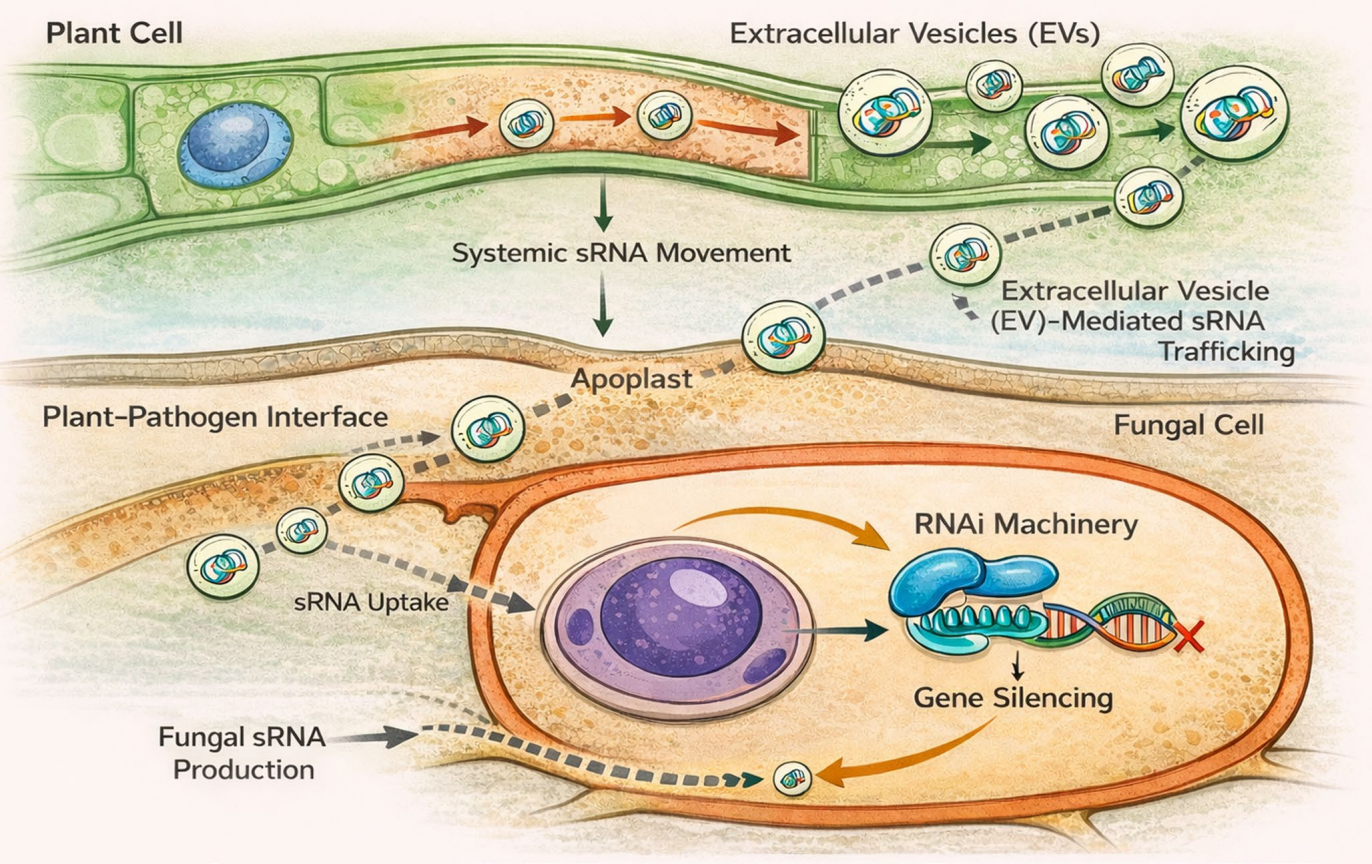
Systemic and cross-kingdom trafficking of sRNAs during plant-pathogen interactions. Plant sRNAs, including miRNAs and siRNAs, can move systemically through vascular tissues such as the phloem and act as long-distance signaling molecules. Some sRNAs are packaged into extracellular vesicles (EVs) and secreted into the apoplast. At the plant-pathogen interface, EVs carrying sRNAs are taken up by fungal pathogens. Inside the fungal cell, these sRNAs interact with the fungal RNA interference (RNAi) machinery, guiding Argonaute complexes to complementary fungal mRNAs and causing gene silencing, including repression of virulence genes. Fungal pathogens may also produce sRNAs that influence host gene expression

One of the most remarkable discoveries in plant microbe biology is that sRNAs can move across species boundaries [20, 70–72], enabling direct post-transcriptional communication between hosts and their interacting organisms. This phenomenon, known a**s** Cross-kingdom RNA interference (ckRNAi) (Fig. 2), has redefined our understanding of molecular dialogue in plant pathogen interactions, revealing that both plants and their pathogens deploy mobile RNAs as offensive and defensive tools in an evolutionary arms race. A seminal study demonstrated that *Botrytis cinerea* produces a suite of sRNAs (Bc-sRNAs) that enter plant cells and hijack the host RNA silencing machinery [20]. Subsequent studies revealed bidirectional RNA trafficking between plants and pathogens, emphasizing the broader role of cross-kingdom RNA interference in plant-microbe interactions [19, 49, 73].

These fungal sRNAs associate with *Arabidopsis* ARGONAUTE1 (AGO1) and suppress the expression of key immunity genes *MPK1*, *MPK2*, and *PR2* [20, 74]. Mutants of *B. cinerea* lacking *DCL1* and *DCL2* enzymes required for sRNA biogenesis were defective in producing these sRNAs and exhibited markedly reduced virulence on both *Arabidopsis* and tomato. Arabidopsis also delivers sRNAs into *B. cinerea* as part of its immune defense [21, 75]. Host cells were found to secrete extracellular vesicles (EVs), membrane-bound structures enriched in tetraspanin proteins (TET8/TET9) that accumulate at infection sites. These vesicles carry a selective cargo of dozens of host-derived sRNAs, many of which target fungal genes required for pathogenicity, such as *Bc-VPS51*, *Bc-DCL1*, and *Bc-DCL2*. Once internalized by the fungus, these transferred sRNAs trigger RNA silencing of their complementary targets, weakening fungal virulence. The process is strikingly selective, that is, specific classes of 21-nt sRNAs are preferentially loaded into EVs, suggesting an active sorting mechanism for defensive RNA cargo.

Similar strategies have since been observed in diverse plant-fungal systems. In cotton [19], demonstrated that *Gossypium hirsutum* exports two miRNAs, miR166 and miR159, into the vascular wilt fungus *Verticillium dahliae*. During infection, these miRNAs were upregulated in cotton and selectively transferred into the fungal hyphae, where they silenced virulence-associated genes encoding a Ca²⁺-dependent cysteine protease (*Clp-1*) and an isotrichodermin C-15 hydroxylase (*HiC-15*). Functional assays confirmed that *V. dahliae* strains expressing miRNA-resistant versions of these genes regained full virulence, demonstrating the specificity and effectiveness of host-delivered RNA silencing.

In cereals [73], showed that infections by *Fusarium graminearum*, the causal agent of Fusarium head blight, were strongly suppressed when double-stranded RNAs targeting the fungal *CYP51* genes were either expressed in planta or applied exogenously. The study revealed that fungal cells can efficiently uptake dsRNAs, process them via DCL1-dependent pathways, and undergo gene silencing, thereby establishing the foundation for Spray-Induced Gene Silencing (SIGS) as a transgene-free antifungal strategy. Furthermore [76], found that HIGS operates even against obligate biotrophic fungi. Transgenic barley expressing double-stranded RNAs directed against the powdery mildew fungus *Blumeria graminis* effector gene *Avra10* exhibited markedly reduced fungal growth. Infection was restored only when the fungus expressed an RNAi-resistant version of *Avra10*, demonstrating that host-derived RNAs can cross the haustorial interface and silence fungal virulence genes.

In other studies, *F. graminearum* exported sRNAs that suppress *barley chitinase* and *superoxide dismutase* genes, while *Phytophthora infestans*,* the causal agent of potato late blight*, delivered small RNA effectors into potato to repress genes involved in hormone signaling and basal defense [21]. Even parasitic plants engage in cross-species RNA trafficking. The dodder, *Cuscuta campestris*, produces numerous 22-nucleotide miRNAs within its haustorial feeding structures during parasitism of *Arabidopsis* [49]. These parasite-derived miRNAs enter host tissues and cleave specific host mRNAs involved in defense and nutrient metabolism, triggering secondary siRNA amplification and sustained transcript silencing. Loss-of-function mutations in host target genes increased parasite biomass, demonstrating that these miRNAs act as trans-species regulators and virulence factors that promote parasitic success. Notably, the same *C. campestris* miRNAs were active in multiple host species, indicating a conserved strategy for broad-spectrum manipulation of host gene expression [49].

The mechanism by which sRNAs traverse cell walls and membranes during cross-kingdom communication has only recently begun to be elucidated. Plants selectively package specific sRNAs and, in some cases, mRNAs into extracellular vesicles (EVs) derived from multivesicular bodies. These EVs are released into the apoplast, where they accumulate at pathogen contact sites and can fuse with the plasma membrane of invading fungal hyphae. Proteomic analyses of *Arabidopsis* EVs revealed enrichment in tetraspanin proteins (TET8, TET9), stress-associated chaperones (HSP70s), and RNA-binding proteins including ARGONAUTE 1 (AGO1), RNA helicases (RH11, RH37), and annexins (ANN1, ANN2) [48, 77]. These RNA-binding proteins confer remarkable cargo selectivity: AGO1, RH11, and RH37 preferentially bind EV-enriched sRNAs, while ANN1 and ANN2 interact more broadly, facilitating sRNA stabilization and export. *Arabidopsis* mutants that have lost the function for *ago1*, *rh11 rh37*, *ann1 ann2* showed reduced secretion of sRNAs in EVs and heightened susceptibility to *B. cinerea*, confirming that EV-mediated RNA export contributes directly to plant immunity [48]. Reciprocally, *B. cinerea* itself uses a parallel EV-based system to deliver fungal sRNAs into plant cells. The fungus secretes tetraspanin-positive EVs marked by the protein Punchless 1 (BcPLS1), which carry virulence-associated sRNAs. These fungal EVs are internalized by *Arabidopsis* through clathrin-mediated endocytosis (CME), a process dependent on the host CLATHRIN LIGHT CHAIN 1. Disruption of CME components significantly reduces *B. cinerea* infection and attenuates sRNA loading into plant AGO1 complexes, leading to diminished suppression of host immunity genes [78].

The bidirectional movement of sRNAs reframes host-pathogen communication as a molecular arms race. Plants produce mobile miRNAs and siRNAs that target fungal virulence factors, while pathogens counter by generating their own sRNAs to suppress host immunity. Comparative genomic and functional studies reveal that the RNA interference (RNAi) machinery of fungi has undergone extensive diversification. Analyses of 43 fungal genomes by [79] showed that the core RNAi components, Dicer and Argonaute are broadly conserved across Basidiomycota and other fungal lineages, but vary greatly in copy number. Ancient duplications followed by lineage-specific gains and losses have produced multiple paralogs in some taxa, whereas others have lost the pathway entirely. Such diversification implies that RNAi systems have been repeatedly molded to meet different ecological and biological demands.

Functional studies in the entomopathogenic fungus *Metarhizium robertsii* further illustrate this adaptive specialization; two Dicer-like genes (*Mrdcl1*, *Mrdcl2*) and two Argonautes (*Mrago1*, *Mrago2*) perform distinct roles, with MrDCL2 and MrAGO1 constituting the primary RNA-silencing machinery required for conidiation and the biogenesis of microRNA-like sRNAs [80]. Loss of these genes markedly reduced fungal sporulation and altered the expression of numerous sRNAs, demonstrating that RNAi not only contributes to genome defense but also regulates fungal development and virulence. In contrast, some obligate biotrophs such as *Ustilago maydis*,* the corn smut fungus*, have entirely lost the core RNAi components, Dicer and Argonaute. Comparative studies between *U. maydis* and its close relative *U. hordei* revealed that only the latter retains a functional RNAi pathway capable of producing siRNAs that mediates gene silencing, whereas *U. maydis* fails to initiate silencing despite the presence of double-stranded RNA substrates [81]. This genomic and functional loss is likely due to an evolutionary trade-off; by dispensing with RNAi, *U. maydis* may have reduced its transposon content while simultaneously avoiding recognition or interference by host-derived sRNAs. The absence of an active RNAi system in this biotroph is a good example of how fungal lifestyles and host interactions can shape the maintenance or elimination of silencing machinery.

These discoveries have also led to the development of RNA-based disease control strategies that exploit the mobility and regulatory functions of small RNAs.

### Hormone-regulated systemic RNA mobility and long-distance defense signaling

Classical systemic acquired resistance (SAR) relies on hormonal messengers such as salicylic acid (SA), jasmonic acid (JA), and ethylene (ET) to propagate immune signals from local infection sites to distal organs [82–84]. More recently, systemic acquired silencing (SAS) mediated by mobile sRNAs has emerged as a parallel signaling system that complements hormonal pathways to achieve coordinated whole-plant immunity [50, 85]. Growing evidence indicates that these pathways intersect, hormonal signaling alters the expression and processing of defense-associated sRNAs, while sRNAs, in turn, fine-tune hormone-responsive transcription factors to balance growth and immunity. The bacterial elicitor flg22, perceived by the pattern-recognition receptor FLS2, rapidly induces miR393, which targets the auxin receptors TIR1 and AFB2. This action represses auxin signaling and in turn enhances basal resistance against *P. syringae* [17]. JA and its methylated derivative (MeJA) are major regulators of wound and necrotrophic pathogen responses. Another study showed that **miR319** modulates **JA biosynthesis** indirectly by repressing TCP transcription factors that activate the lipoxygenase gene *LOX2*, a key enzyme in JA production [86]. Conversely, exogenous MeJA treatment alters the expression of numerous miRNAs associated with stress and defense, indicating that JA signaling can in turn regulate miRNA transcription and turnover [87, 88]. Although small RNAs share features with classical hormones, such as mobility and systemic effects, they differ mechanistically as they lack dedicated receptors and act through sequence-specific gene silencing pathways.

These reciprocal interactions between hormonal signaling and sRNAs illustrate how local defense activation can contribute to the establishment of systemic immunity. Hormonal messengers and RNA-based silencing signals work together to coordinate whole-plant defense responses. Molecules such as salicylic acid (SA), methyl salicylate, and azelaic acid act as mobile cues that prime distal tissues through redox and chromatin changes, while mobile siRNAs generated at infection sites travel through the phloem to enforce post-transcriptional or transcriptional gene silencing in recipient cells [56, 85, 89]. The cooperation between hormone-driven transcriptional reprogramming and sRNA-mediated silencing establishes a multilayered systemic defense network capable of both rapid induction and lasting reinforcement. Genetic and physiological studies support this functional convergence. Components of the RNAi machinery, including DICER-LIKE and ARGONAUTE proteins, are required for full immune competence, and their loss leads to compromised resistance to bacterial and viral pathogens [90, 91]. Conversely, hormonal priming enhances the expression of several RNA silencing components and sRNA populations associated with systemic defense [85]. The convergence of hormonal and RNA-mediated signaling likely represents an evolutionary optimization that allows plants to balance defense strength with metabolic economy. By linking phytohormone perception to RNA mobility, plants ensure that systemic defense signaling is context-dependent, robust under pathogen challenge yet reversible under normal growth. Understanding how hormones influence RNA mobility could thus inform new strategies for engineering durable yet energy-efficient immunity in crops.

Despite these advances, several biological and technical challenges remain in understanding and applying systemic RNA signaling in plants.

### Unanswered questions and limitations

Although substantial progress has been made in establishing systemic RNA signaling in plants, several important unanswered questions remain. The movement of small RNAs between distant tissues is not always consistently observed, which may result from differences in experimental design, detection sensitivity, or RNA stability [18, 37, 92]. For example, grafting experiments in *A. thaliana* have demonstrated RNA movement within the plant, but restricted mobility has also been reported depending on tissue type or developmental stage [37, 92]. In addition, the apparent mobility of small RNAs may, in some cases, reflect passive transport through the phloem rather than regulated signaling [30, 52]. Furthermore, the functional contribution of these small RNAs to defense responses remains difficult to resolve, as their origin is often ambiguous. While trans-acting and secondary siRNAs contribute to systemic silencing, they cannot always be distinguished from amplification products of local responses [28, 93].

Cross-kingdom RNA interference (ckRNAi) provides functional evidence for systemic RNA movement, as demonstrated by the delivery of small RNAs from *Arabidopsis* and cotton to the fungal pathogen *B. cinerea*, as well as HIGS-based resistance in wheat against *F. graminearum* [20, 73]. However, this mechanism appears to be more applicable to fungal interactions than to bacterial or viral systems. In bacterial infections, there is currently limited evidence supporting small RNA-mediated inter-kingdom communication, likely due to differences in uptake mechanisms and the absence of canonical RNA interference machinery in bacteria [94]. In the case of viruses, which both trigger and suppress RNA silencing pathways, the systemic movement and functional contribution of host-derived small RNAs remain incompletely understood, partly due to virus-encoded silencing suppressors [94]. Finally, most available data derive from model organisms such as *A. thaliana* and *N. benthamiana*, and their relevance to crop systems under field conditions remains to be fully established [20, 95]. Although SIGS and HIGS show promise in crops including barley, wheat, and maize, their effectiveness is strongly influenced by environmental conditions, and efficient delivery remains a major challenge. Consequently, conclusions drawn from model systems should be interpreted with caution and require further validation in agriculturally relevant species.

### Future perspectives

Despite substantial progress, the field faces several unresolved questions that will define the next phase of RNA-based plant science. At the mechanistic level, a complete understanding of how sRNAs are selected for export, packaged into EVs or ribonucleoprotein complexes, and recognized by recipient cells remains elusive [48, 53]. Identifying the protein and lipid components that govern cargo selectivity, membrane fusion, and cross-kingdom entry will be crucial to manipulate RNA movement with precision. Furthermore, the balance between RNA amplification and degradation during long-distance transport is poorly characterized [85], leaving open questions about how plants maintain systemic silencing without compromising developmental or metabolic functions. At the translational level, bringing RNA-based protection from controlled experiments to the field will require coordinated advances across disciplines. The variability of RNA uptake among pathogens, the instability of unprotected RNAs in environmental conditions, and barriers to efficient tissue penetration continue to restrict efficacy [22, 96]. Overcoming these limitations will require combining materials science, for the creation of biodegradable carriers that extend RNA stability [97], with synthetic biology, to engineer plant systems that express and deploy double-stranded RNAs with spatial and temporal precision [98].

Beyond experimental innovation, bioinformatics and data integration remain a major bottleneck. Current sequencing pipelines and small RNA annotation tools struggle to distinguish true mobile RNAs from abundant endogenous fragments, often underrepresenting low-copy or condition-specific species [23]. Developing standardized computational workflows that integrate small RNA, degradome, and vesicle proteomic data will be essential to identify functional mobile RNAs and predict their targets with accuracy. Complementary AI-driven pipelines could further enhance design by modeling degradation kinetics, secondary structure, and host-pathogen specificity [99, 100]. Equally important are the ecological and regulatory dimensions of RNA-based biocontrol. Although current data indicate that dsRNAs degrade rapidly in soil and show negligible trophic transfer, systematic field-scale assessments remain scarce [101]. The ecological effects on beneficial microbiota, pollinators, and non-target arthropods must be quantified to ensure biosafety and public acceptance. Establishing harmonized regulatory frameworks akin to those used for microbial or peptide biopesticides will be essential for risk assessment, product registration, and market adoption [102].

## Conclusion

In summary, growing evidence supports a central role for small RNAs (sRNAs) in systemic signaling during plant defense. Both miRNAs and siRNAs contribute to long-distance immune communication and cross-kingdom interactions, highlighting the bidirectional nature of RNA-based regulation between plants and their associated organisms. Despite these advances, key mechanistic questions remain unresolved, particularly regarding the selective loading, transport, and delivery of mobile sRNAs, as well as the identity and functional relevance of the molecules driving systemic immune responses. Future research should focus on elucidating the molecular machinery underlying sRNA mobility and defining the specificity of cross-kingdom RNA trafficking under biologically relevant conditions. Integrating emerging technologies such as high-resolution imaging, RNA tracking, and single-cell sequencing will be critical to resolving these processes. Advancing our understanding of systemic sRNA signaling will not only deepen insight into plant immunity but also support the development of innovative and sustainable RNA-based crop protection strategies.

## Data Availability

No datasets were generated or analysed during the current study.

## Abbreviations

sRNA: Small RNA
miRNA: MicroRNA
siRNA: Small interfering RNA
SAR: Systemic acquired resistance
SAS: Systemic acquired silencing
ckRNAi: Cross-kingdom RNA interference
HIGS: Host-induced gene silencing
SIGS: Spray-induced gene silencing
EVs: Extracellular vesicles
AGO: Argonaute
DCL: Dicer-like
